# Impact of Antibiotic Consumption on Antimicrobial Resistance to Invasive Hospital Pathogens

**DOI:** 10.3390/antibiotics12020259

**Published:** 2023-01-28

**Authors:** Deana Medic, Bojana Bozic Cvijan, Milica Bajcetic

**Affiliations:** 1 Faculty of Medicine, University of Novi Sad, Hajduk Veljkova 3, 21000 Novi Sad, Serbia; 2 Institute of Public Health of Vojvodina, Center for Microbiology, 21000 Novi Sad, Serbia; 3 Department of Pharmacology, Clinical Pharmacology and Toxicology, Faculty of Medicine, University of Belgrade, P.O. Box 38, 11000 Belgrade, Serbia; 4 Clinical Pharmacology Unit, University Children’s Hospital, 11000 Belgrade, Serbia

**Keywords:** antibiotics, resistance, consumption, pathogen

## Abstract

The aim of our investigation is to correlate the wholesale data on antibiotic consumption expressed in daily doses per 1000 inhabitants per day (DID) with the resistance rate of invasive pathogen bacteria from 2017 to 2021. The data on antimicrobial resistance were collected from an analysis of the primary isolates of hospitalized patients. According to the CAESAR manual, the selected pathogens isolated from blood culture and cerebrospinal fluids were tested. The consumption of antibiotics for systematic use showed a statistically significant increasing trend (β = 0.982, *p* = 0.003) from 21.3 DID in 2017 to 34.5 DID in 2021. The ratio of the utilization of broad-spectrum to narrow-spectrum antibiotics increased by 16% (β = 0.530, *p* = 0.358). The most consumed antibiotic in 2021 was azithromycin (15% of total consumption), followed by levofloxacin (13%) and cefixime (12%). A statistically positive significant correlation was discovered between the percentage of resistant isolates of *K. pneumoniae* and consumption of meropenem (r = 0.950; *p* = 0.013), ertapenem (r = 0.929; *p* = 0.022), ceftriaxone (r = 0.924; *p* = 0.025) and levofloxacin (r = 0.983; *p* = 0.003). Additionally, the percentage of resistant isolates of *E. coli* and consumption of ertapenem showed significant correlation (r = 0.955; *p* = 0.011). Significant correlation with consumption of the antibiotics widely used at the community level, such as levofloxacin, and resistance isolated in hospitals indicates that hospital stewardship is unlikely to be effective without a reduction in antibiotic misuse at the community level.

## 1. Introduction

Excessive antibiotic use, among other factors, is a major contributor to the development of antimicrobial drug resistance (AMR) [1]. It has been estimated that in 2019, nearly 5 million deaths were associated with bacterial antimicrobial resistance, including 1.27 million deaths directly attributable to such resistance [2]. The “silent pandemic” synonym for AMR became even more pronounced during the COVID-19 pandemic and ultimately led to even more deaths [3]. 

As a result of growing concerns, several activities were launched in Serbia over the last decade with the aim of reducing AMR rates. A pioneer step was the increased control of pharmacies in 2011 in order to stop the dispensing of antibiotics without prescription. In 2015, the first campaign for rational antibiotic use was launched by the Serbian Ministry of Health and implemented through the Second Serbia Health Project funded by the World Bank. After the campaign, the trend of decreased antibiotic consumption became evident: the data for 2016 and 2017 showed a significant decrease compared to 2015 by a total of 32.79% [4]. In addition, good insight into the prevalence of hospital-acquired infections (HAI) as well as the most frequent causative pathogens in Serbian hospitals was provided by the European point prevalence surveys (PPS) on HAI and antimicrobial use in acute care hospitals, organized by the European Centre for Disease Prevention and Control (ECDC) [5,6]. Although Serbia is not part of the European Union (EU), we have participated in all PPS from 1998, including the current one in 2022. Further efforts to apply the latest knowledge regarding appropriate antimicrobial use were made in 2018 when a guideline for rational antibiotic use was published [7]. 

In addition to the World Health Organization (WHO) Europe Antimicrobial Medicines Consumption (AMC) Network, Serbia is the part of the Central Asian and European Surveillance (CAESAR) network. The system for the registration and monitoring of AMR consists of the National Reference Laboratory for Registration and Monitoring of Bacterial Strains Resistance to Antimicrobial Agents and the national network of 24 clinical laboratories, established on a voluntary basis. The susceptibility of invasive isolates from blood and cerebrospinal fluid to antibiotics is monitored using the same methodology used by EU countries, in accordance with the applicable international standards [8]. The data regarding AMC and bacterial resistance from Serbia are part of the annual WHO Europe AMC and CAESAR reports [4,8]. 

An unexpected obstacle in combating AMR was caused by the COVID-19 pandemic. Despite the lack of evidence to support azithromycin or prescribing antibiotics in general, clinicians all over the world are using it to treat COVID-19 patients [9,10]. A recently published study showed that almost three quarters of COVID-19 patients have received antibiotics and more than a half have received an IV antibiotic [11,12]. The COVID-19 pandemic made combating AMR an even greater challenge. 

The aim of the study was to investigate the relationship between wholesale data of antibiotic consumption and invasive bacteria resistance rate in the Republic of Serbia (RS) from 2017 to 2021.

## 2. Results

The total consumption of antibiotics for systemic use (J01 group) increased from 21.3 DID in 2017 to 34.5 DID in 2021 (Table 1). The overall observed increasing trend in total antibiotic consumption between 2017 and 2021 was statistically significant (β = 0.982, *p* = 0.003). 

The quality indicators describing the relative use of beta-lactamase-sensitive penicillin (J01CE), penicillin, including beta-lactamase inhibitor (J01CR), third- and fourth-generation cephalosporins (J01DD + J01DE), carbapenems (J01DH) and fluoroquinolones (J01MA), as well as the ratio of utilization of broad-spectrum to narrow-spectrum penicillin, cephalosporins and erythromycin (J01 B/N) in Serbia from 2017 to 2021 are shown in Table 1.

A statistically significant decrease in the share of narrow spectrum penicillin was observed from 2017 to 2021 (β = −0.979, *p* = 0.004). A decreasing trend, although not statistically significant, was observed in the share of penicillin, including beta-lactamase inhibitor (β = −0.018, *p* = 0.978). Opposite to the trend of penicillin use is the statistically significant increasing trend of the share of third- and fourth-generation cephalosporins (β = 0.882, *p* = 0.047). The trend of increased carbapenemes consumption was observed (β = 0.847, *p* = 0.070). Fluoroquinolones were the most commonly used antibiotic pharmacological subgroup, with an overall increasing trend (β = 0.618, *p* = 0.267). The ratio of the utilization of broad-spectrum to narrow-spectrum penicillin, cephalosporins and macrolides (J01_B/N) increased over the period 2017–2021 (β = 0.530, *p* = 0.358).

The consumption of beta-lactamase sensitive penicillin (J01CE) expressed as a percentage of the total consumption of antibacterials for systemic use (J01), i.e., relative consumption of beta-lactamase sensitive penicillins J01CE_%; consumption of combination of penicillin, including beta-lactamase inhibitor (J01CR), expressed as a percentage of the total consumption of antibacterials for systemic use (J01), i.e., relative consumption of a combination of penicillin, including beta-lactamase inhibitor J01CR_%; consumption of third- and fourth-generation cephalosporins (J01(DD + DE)) expressed as percentage of the total consumption of antibacterials for systemic use (J01), i.e., relative consumption of third- and fourth-generation cephalosporins J01DD + DE_%; consumption of carbapenems (J01DH) expressed as a percentage of total consumption of antibacterials for systematic use (J01); consumption of fluoroquinolones (J01MA) expressed as a percentage of the total consumption of antibacterials for systemic use (J01), i.e., relative consumption of J01MA_%; ratio of the consumption of broad-spectrum B (J01(CR + DC + DD+(F-FA01)) to the consumption of narrow-spectrum penicillin, cephalosporins and erythromycin (J01(CE + DB + FA01)) J01_B/N%.

**Figure 1:**
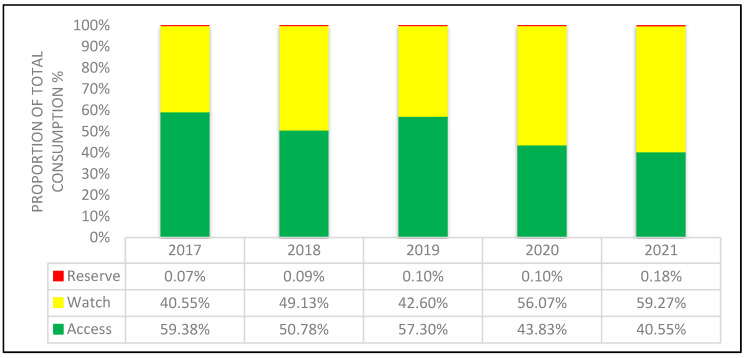
Total consumption of antibiotics for systemic use according to WHO AWaRe classification, percentage by class from 2017 to 2021.

The percentage of the total antibiotic consumption of Access, Watch and Reserve group antibiotics is shown in Figure 1. The total consumption of Access antibiotics decreased from 59.38% in 2017 to 40.55% in 2021. The WHO national monitoring target of at least 60% of total antibiotic consumption coming from Access agents was not met during the period of time studied. The total consumption of Watch antibiotics increased from 40.55% in 2017 to 59.27% in 2021. The WHO national monitoring target of a maximum of 35% of total antibiotic consumption coming from Watch agents was exceeded in each monitored year, reaching as much as 59.27% in 2021. The total consumption of Reserve antibiotics was low in all analyzed years.

The amoxicillin index decreased from 20% in 2017 to 11% in 2021 (Table 2).

The ten most consumed antibiotics for systemic use in Serbia represent 80% and more of the total consumption: 80% in 2017, 83% in 2018, 84% in 2019, 86% in 2020 and 85% in 2021. During the period studied, the pattern and quantity of the most frequently consumed antibiotics changed significantly. The most commonly consumed antibiotic in Serbia in 2017, 2018 and 2019 was amoxicillin (20%, 18% and 19% respectively). In 2020 and 2021, azithromycin was the most commonly used antibiotic (21%, 15%). Levofloxacin was the 7th most commonly used antibiotic in 2017, 2018 and 2019; 4th in 2020 and 2nd in 2021. In addition to levofloxacin, increased consumption during the analyzed years is demonstrated for cefixime, the 3rd most commonly used antibiotic in 2021 (Table 3). In addition, one of the highest increases in consumption was observed for meropenem 415% (from 0.035 DID in 2017 to 0.148 in 2021), which was the 25th most commonly used antibiotics in 2021, and ertapenem 206% (from 0.009 DID in 2017 to 0.020 in 2021).

In this study, the total number of isolated strains was as follows: 2336, 2811, 2895, 2509, 3740 in 2017, 2018, 2019, 2020 and 2021, respectively. The total number of isolates of *Acinetobacter* spp., *Klebsiella pneumoniae* (*K. pneumoniae*) and *Enterococcus* spp. increased from 2017 to 2021. A decreasing trend was observed for the isolates of *Escherichia coli* (*E. coli*), *Pseudomonas aeruginosa (P. aeruginosa)*, *Staphylococcus aureus* (*S. aureus*) and *Streptococcus pneumoniae* (*S. pneumoniae*).

The highest percentage of multidrug-resistant (MDR) isolates of over 90% was present in *Acinetobacter* spp. isolates, 70% in *K. pneumoniae* and 50% in *P. aeruginosa*, while a negative trend was recorded in *E. coli* during all five years. MDR trend movements over the relevant observed period are not statistically significant for any bacteria (*K. pneumoniae*, *E. coli*, *P. aeruginosa*, *Acinetobacter* spp.) (Table 4).

### Correlation between Antibiotic Consumption and Resistance Rate

**Figure 2:**
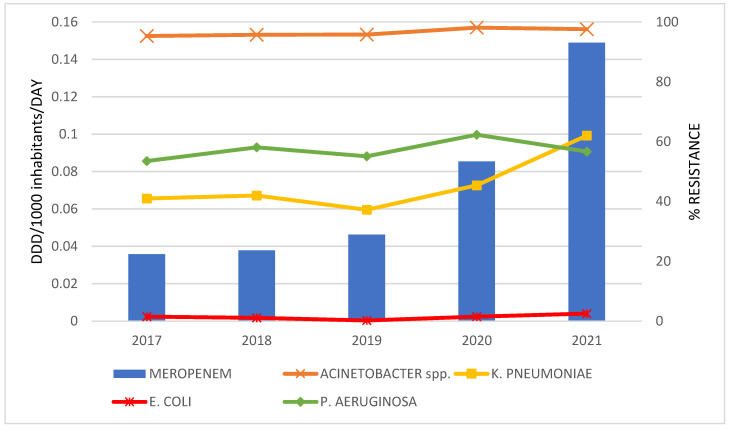
The correlation of meropenem consumption and the resistance to meropenem in bacteria during 2017–2021.

**Figure 3:**
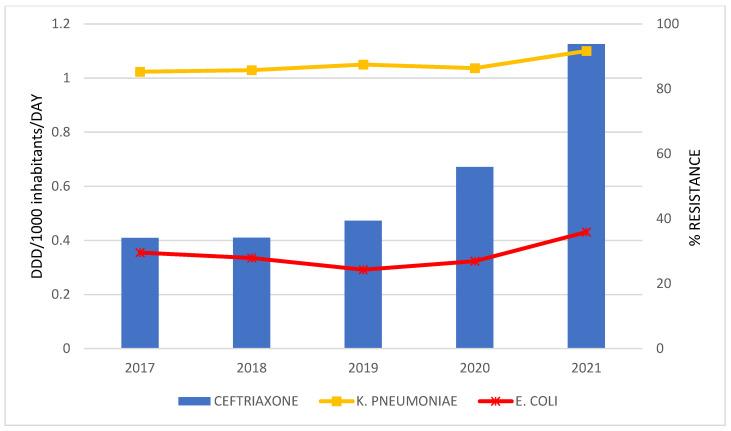
The correlation of ceftriaxone consumption and the resistance to ceftriaxone in bacteria during 2017–2021.

**Figure 4:**
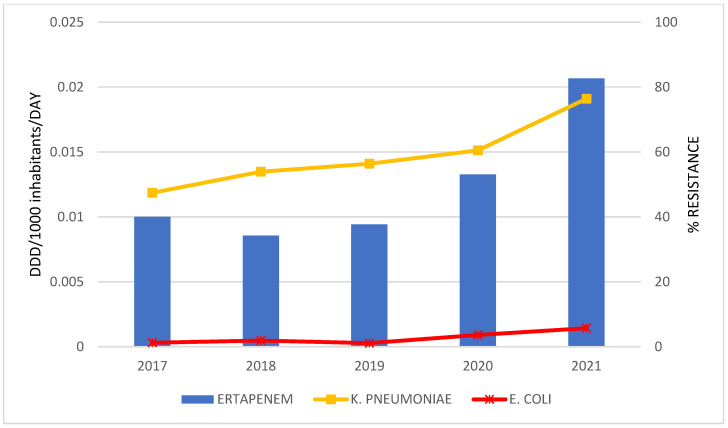
The correlation of ertapenem consumption and the resistance to ertapenem in bacteria during 2017–2021.

**Figure 5:**
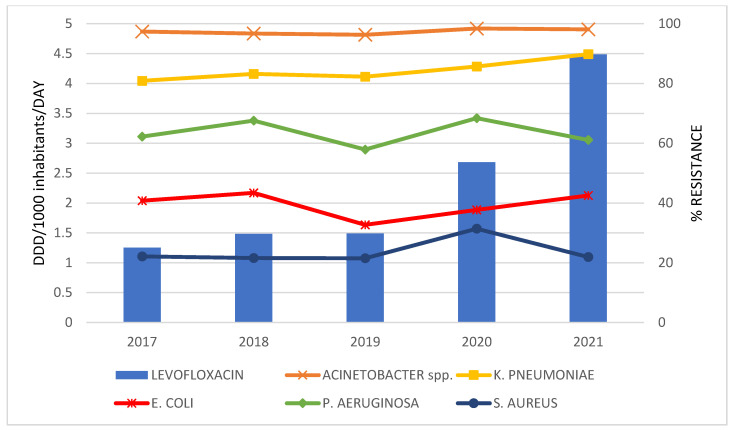
The correlation of levofloxacin consumption and the resistance to levofloxacin in bacteria during 2017–2021.

The correlations between antibiotics consumption and the resistance rate of bacteria for the period 2017 to 2021 are graphically presented only in cases where a statistically strong correlation was established (Figure 2, Figure 3, Figure 4 and Figure 5).

A statistically strong correlation was discovered for the consumption of: meropenem and the percentage of resistant isolates of *K. pneumoniae* (r = 0.950; *p* = 0.013); ceftriaxone and *K. pneumoniae* (r = 0.924; *p* = 0.025); ertapenem and *K. pneumoniae* (r = 0.929; *p* = 0.022) and *E. coli* (r = 0.955; *p* = 0.011); levofloxacin and *K. pneumoniae* (r = 0.983; *p* = 0.003).

Meanwhile, a negative correlation was observed between the consumption of: colistin and *Acinetobacter* spp. (r = −0.804; *p* = 0.101); ceftazidime and *E. coli* (r = −0.540; *p* = 0.347), imipenem and *Acinetobacter* spp. (r = −0.121; *p* = 0.846) and *P. aeruginosa* (r = −0.114; *p* = 0.856); gentamicin and *Acinetobacter* spp. (r = −0.394; *p* = 0.511), *E. coli* (r = −0.279; *p* = 0.649); linezolid and *Enterococcus* spp. (r = −0.522; *p* = 0.367), *S. aureus* (r = −0.229; *p* = 0.710); ciprofloxacin and *Acinetobacter* spp. (r = −0.349; *p* = 0.564), *P. aeruginosa* (r = −0.250; *p* = 0.685), *S. aureus* (r = −0.337; *p* = 0.579); levofloxacin and *P. aeruginosa* (r = −0.034; *p* = 0.956).

No significant correlation was found for the consumption of: meropenem and *Acinetobacter* spp., *E. coli*, and *P. aeruginosa*; colistin and *K. pneumoniae* and *P. aeruginosa*; amoxicillin clavulanic acid and *K. pneumoniae* and *E. coli*; ceftriaxone and *E. coli*; cefepime and *P. aeruginosa*; ceftazidime and *K. pneumoniae*, *E. coli* and *P. aeruginosa*; imipenem and *K. pneumoniae* and *E. coli*; erythromycin and *S. pneumoniae*; gentamicin and *K. pneumoniae*; ciprofloxacin and *K. pneumoniae* and *E. coli*; levofloxacin and *Acinetobacter* spp., *E. coli* and *S. aureus*; vancomycin and *Enterococcus* spp..

## 3. Discussion

To the best of our knowledge, this is the first study correlating antibiotic consumption and antimicrobial resistance rate of hospital pathogens in Serbia over a five-year period, from 2017 to 2021. In order to comprehend the current status of antibiotics usage in the RS, qualitative and quantitative changes in DID and qualitative indicators of most frequently used antibiotics were carefully monitored.

During the monitored period, the utilization of antibiotics increased from 21.3 DDD to 34.5 DDD per 1000 inhabitants per day from 2017 to 2021, respectively. The highest increase was observed during the COVID-19 pandemic in 2020 and 2021. The clear pattern in the rise in antibiotic consumption during the COVID-19 pandemic is not unique for our country [13]. Nevertheless, a significantly higher number of isolated strains should be taken into account when interpreting these results. We assume that the significant decrease in antibiotic consumption (by one third from 2016 to 2018), which is the result of a government campaign, significantly lessened the expected increase in antibacterial resistance due to the excessive antibiotic consumption observed during the COVID-19 years [4]. This is supported by the result of our research which reveals no increased trend in MDR isolates of *K. pneumoniae*, *E. coli*, *P. aeruginosa* and *Acinetobacter* spp. over the observed period, including the COVID-19 years.

Furthermore, we not only identified variations in the rates of total antibiotic consumption, but also in the qualitative proportion of used antibiotics. One major concern is the trend of increasing consumption of broad- vs narrow-spectrum antibiotics (J01 B/N) over a 5-year period in RS. Our result is similar to the findings of the trend of high consumption of broad-spectrum antibiotics observed in neighboring countries such as Bulgaria, Croatia, Hungary and Slovenia as well as Latvia over the last ten years [14]. North European countries such as Ireland, Iceland and Finland showed the most significant decreasing trend in the consumption of the broad-spectrum antibiotics [14]. These findings can be identified as targets for further improvement strategies for antibiotic consumption reduction and allow an exchange of knowledge with countries with the lowest consumption of broad-spectrum antibiotics.

AWaRe categories were observed during the analyzed period of time as an important quality indicator of antibiotic consumption [15]. Trends in the use of AWaRe categories were found to change significantly when comparing 2017 to 2021. During the five-year period, the total consumption of Access antibiotics decreased from 59.38% to 40.55%. According to the published data, of all the members in the AMC network, only four and five countries have met the WHO target of at least 60% of total consumption in regard to Access agents in 2017 and 2018, respectively [4].

Watch group antibiotics accounted for 40.6%, 49.3%, 42.6%, 56% and 59.3% in 2017, 2018, 2019, 2020 and 2021, respectively. In 2021, in 9 out of 28 European countries (Norway, Malta, Greece, Hungary, Romania, Cyprus, Italy, Slovakia and Bulgaria), the proportion of Watch antibiotic use was higher than 40% [14]. The total consumption of Watch antibiotics ranged from 13% in Iceland to 61% in Slovakia of the total consumption for the ESAC-Net countries over the period from 2014 to 2018 [16]. Over the same period of time, the total consumption of Watch antibiotics ranged from 34% in Bosnia and Herzegovina to 69% in Uzbekistan for the WHO Europe AMC Network countries [4]. The globally observed high levels of consumption of Watch agents represent the target for future initiatives for qualitative improvement in rational antibiotic prescribing [16]. In RS during 2021, antibiotics from the Watch group comprised 53% of the 10 most commonly prescribed antibiotics: azithromycin, levofloxacin, cefixime, clarithromycin, ceftriaxone. These findings are particularly worrisome due to the possible link between consumption of these antibiotics and bacterial resistance development [17]. Another important result has been noted in the qualitative changes in the ten most commonly consumed antibiotics in RS during the monitored period: a significant upward trend has been noted for azithromycin, levofloxacin and cefixime; all representatives of the Watch antibiotic group [15]. Similar upward findings were noted in China, India, as well as in the majority of low-income countries [18,19]. The increase in consumption of Watch antibiotics worldwide can be explained by the greater affordability of expensive broad-spectrum antibiotics, the specificity of local pharmaceutical markets, weak regulatory capacities, uncertain diagnostic tests for febrile illnesses, prevention of AMR being low priority, and a higher rate of infections resistant to antibiotics from the Access group [18]. The Reserve group antibiotics remained within the recommended scope (significantly below 5%), with an obvious increasing trend. None of the antibiotics from the Reserve group were among the 10 most commonly prescribed antibiotics during the monitored period of time.

Additionally, as an important qualitative indicator, the amoxicillin index was calculated. In RS, the amoxicillin index fell from 17.5% to 11.4% from 2017 to 2021. Globally, the amoxicillin index varied from 38.7% in Tunisia to the lowest one, in Kuwait of 1.9% [18]. As previously reported, due to the frequent unavailability of narrow-spectrum penicillin in Serbia [20], the percentage of their total consumption is negligible. The decreasing trend in the consumption of amoxicillin is parallel with increased azithromycin consumption and can be attributed to changed prescribing policies caused by the COVID-19 pandemic. Namely, a study with a small number of patients infected with COVID-19 set a hypothesis of viral reduction when azithromycin was used with hydroxychloroquine [21]. All the consequences of this widely accepted hypothesis are yet to be discovered.

### Correlation between Antibiotic Consumption and Bacterial Resistance Rates

Our data demonstrated that the percentage of resistant isolates of *K. pneumoniae* had a significant correlation with the consumption of meropenem, ceftriaxone, ertapenem and levofloxacin. Although there is no statistically significant trend of growth, over 50 to 71% of total *K. pneumoniae* isolates were MDR over the last five years in Serbia. Our findings are in line with the data from the surrounding countries (Greece, Bulgaria, Romania, Bosnia and Hercegovina). The MDR isolate rates vary by region. The lowest rate of MDR *K. pneumoniae* isolates was observed in Scandinavian countries [22]. Furthermore, the consumption of ertapenem was significantly correlated with the percentage of resistant isolates of *E. coli*. Unlike with *K. pneumoniae*, *Acinetobacter* spp., and *P. aeruginosa*, the percentage of MDR *E. coli* isolates were low up to a total of 20% during the observed period.

*K. pneumoniae* is a member of the so called ESKAPE (*Enterococcus faecium, S. aureus, K. pneumoniae, Acinetobacter baumannii, P. aeruginosa, Enterobacter* species) bacteria, common causes of life-threatening nosocomial infections, characterized by multiple drug resistance mechanisms [23]. Several studies described a positive association between bacterial resistance and antibiotic consumption [24,25,26,27,28]. Additionally, the link between decreased antibiotic consumption and the reduction in antibiotic resistance rates has also been reported [26,29]. A possible explanation for these results is in the following sequence of the events. Third-generation cephalosporins were commonly prescribed antibiotics, especially towards the end of our study. So far, their increased use has led to the higher prevalence of extended-spectrum beta-lactamase and, consequently, a higher proportion of prescribed carbapenem antibiotics [27]. Increased consumption of carbapenem leads to the production of carbapenemases (KPC, New Delhi metallo-β-lactamase-1, oxacillinase). As a final impact, an increased rate of *K. pneumoniae* can be noted [23]. The positive correlation between fluoroquinolones consumption and *K. pneumoniae* contributed to the transferable plasmid in extended-spectrum beta-lactamase strains that led to fluoroquinolone resistance [30]. In addition to the transfer of resistant plasmids, multiple resistance mechanisms of *K. pneumoniae* to fluoroquinolones were shown: changes in target sites and changes in outer membrane protein permeability and the efflux pump [31].

Another worrisome piece of data is the result that *E. coli,* followed by *K. pneumoniae,* was responsible for the most deaths attributable to antimicrobial resistance in 2019 [2]. Mounting evidence demonstrates the positive association of carbapenem use and increased rate of *E. coli* resistance [25,31,32,33]. Due to the restricting management options for clinicians, an inevitable conclusion is the need for a rationalization of carbapenem consumption. Additionally, further research aimed at obtaining information about antibiotic resistant genes in MDR bacteria is essential for antibiotic use recommendations. The availability of novel antimicrobial drugs on the market is very important, but it only represents an interim solution for the very complicated AMR situation. Unfortunately, the wide use of novel antibiotics (e.g., ceftazidime/avibactam, imipenem/relebactam) has already accelerated resistance to them and made them useless for some MDR bacteria treatment in a very short time [34,35]. Finally, antimicrobial stewardship programs including the computerized tools for clinical decision support systems should be broadly implemented to increase appropriate antibiotic use and improve the quality of infection care.

Although our analysis of five-year trends in antibiotic consumption has important policy implications, the study has limitations that need to be mentioned. The limitations of the study are unmeasured factors, such as disease conditions, coexisting comorbidities, combined medication use, and the average number of hospitalization days of patients that may influence the results concerning antibiotic consumption and the bacterial resistance rate.

## 4. Materials and Methods

In this study, we have used data on antibiotic consumption for systemic use (J01) based on wholesale data in Serbia generated for annual WHO AMC network reports as the national focal point over the period from January 01, 2017 to December 31, 2021. Consumption data are based on the WHO ATC/DDD (Anatomical Therapeutic Chemical/Defined Daily Dose) methodology, as well as on the WHO Access, Watch and Reserve (AWaRe) classification, version from 2021. Antibiotic consumption expressed in DDD per 1000 inhabitants per day (DID) was calculated for the total population, with the source being the Statistical Office of the RS. The methodology of data analysis was described in detail in the Report for 2019 by the WHO Regional Office for Europe AMC Consumption Network, published in 2022 [36].

The data on antimicrobial resistance were collected over the period from 1 January 2017 to 31 December 2021, from the primary isolates of hospitalized patients, i.e., only one isolate per patient was included. These were invasive isolates originating from blood cultures and cerebrospinal fluid. According to the CAESAR manual [37], the following pathogens were observed: *Escherichia coli*, *Klebsiella pneumoniae*, *Pseudomonas aeruginosa*, *Acinetobacter* spp., *Staphylococcus aureus, Enterococcus faecalis*, *Enterococcus faecium* and *Streptococcus pneumoniae*. For testing and interpretating the antimicrobial susceptibility results, the EUCAST standard (European Committee on Antimicrobial Susceptibility Testing) was used [38], which is also recommended by the CAESAR manual. Antibiotic susceptibility testing was performed using the Kirby–Bauer disc diffusion method or automated systems (Vitek 2 or Phoenix system) and, where required by the standard, the minimum inhibitory concentration (MIC) was also determined using gradient tests (Etest). All isolates of a specific bacterial species were tested against all recommended antibiotics according to the CAESAR manual. MDR isolates were defined as bacteria resistant to three or more groups of antibiotics active against an isolated microorganism. CAESAR network in Serbia currently has 24 laboratories, geographically representing a reliable sample for Serbia. The data cover 78% of the population. Each laboratory in the network adheres to the standard principles of resistance surveillance methodology and sends their data for processing to the NRL for AMR (National Reference Laboratory for Antimicrobial Resistance).

### Statistical Analysis

The Statistical Package for Social Sciences—SPSS 21 was used for statistical data processing. Total consumption over the time was presented by linear regression and evaluated using the analysis of variance (ANOVA) test. A linear relationship between two variables was examined using the Pearson correlation coefficient. A linear logit regression analysis was used for the examination of relationships between two or more variables to generate adequate statistical models. P values ≤ 0.05 were considered statistically significant.

## 5. Conclusions

The high proportion of multidrug-resistant strains of *Acinetobacter* spp., *K pneumoniae* and *P. aeruginosa* found in the present study might be the result of the high consumption trend of broad-spectrum antibiotics in Serbia and the region. Our research highlights the positive correlation between the consumption of ertapenem and the resistance rates of two Gram-negative bacteria: *K. pneumoniae* and *E. coli*, mostly responsible for deaths attributable to antimicrobial resistance. The fact that in addition to ertapenem, meropenem, ceftriaxone and levofloxacin also showed a positive correlation with a resistance rate of *K. pneumoniae* is particularly concerning. The positive correlations were observed with antibiotics with the highest increase in consumption during the COVID-19 pandemic for both inpatient and outpatient care, such as levofloxacin and ceftriaxone, as well as ertapenem and meropenem for inpatient care use. In addition, the dramatically changed structure of antibiotics consumption during the COVID-19 era may have also contributed to AMR. The Watch group of antibiotics represented almost 60% of total antibiotic consumption in the last two years. Based on the discussed findings, several measures should be implemented in the fight against AMR in Serbia: improving the availability of phenoxymetilpenicillin on the market and increasing the amoxicillin index; restricting the use of fluoroquinolones according to the European Medicines Agency (EMA) and guideline recommendations; limiting the use of cefixime, cephalosporin of 3rd generation for oral use as the extension of hospital treatment for outpatients; stronger control of pharmacies and more rigorous punishment for dispensing antibiotics without a prescription. Educating clinical staff, as well as introducing them to technologies such as the clinical decision support system for the needs of the hospital stewardship programs, will significantly improve antibiotic prescription practice and resistance monitoring.

## Figures and Tables

**Table 1:** Changes in the quality indicators in Serbia from 2017 to 2021.

Total Consumption of J01 Antibacterials in DDD/1000 Inhabitants per Day						CAGR ^a^	Linear Regression Analysis		
	2017	2018	2019	2020	2021		Β	β	P
Total J01	21.3	22.7	26.6	29.2	34.5	10.13%	3.290	0.982	0.003
J01CE_%	0.3866	0.3340	0.27386	0.13180	0.10214	−23.37%	−0.077	−0.979	0.004
J01CR_%	9.3360	11.2451	13.7970	9.6052	10.0541	1.49%	−0.020	−0.018	0.978
J01DD + DE_%	7.1499	8.2317	7.7851	10.9696	16.3513	17.92%	2.114	0.882	0.047
J01DH_%	0.2759	0.2689	0.2720	0.36904	0.55181	14.87%	0.065	0.847	0.070
J01MA_%	14.8300	15.2420	12.5994	15.6842	19.4388	5.56%	0.966	0.618	0.267
J01_B/N%	10.5190	24.8976	14.4870	21.4297	22.2950	16.22%	2.008	0.530	0.358

CAGR ^a^: compound average growth rate. B: unstandardized coefficients. β: standardized coefficients.

**Table 2:** Changes in quality indicator—amoxicillin index in Serbia from 2017 to 2021.

Components of Amoxicillin Index	DDD/1 000 Inhabitants per Day (% of Total Oral J01)				
	2017	2018	2019	2020	2021
Amoxicillin and phenoxymethylpenicillin	4.251818	3.996629	4.94175	3.457339	3.930705
Total oral J01 consumption	21.33367	22.75571	26.56083	29.15946	34.47042
The amoxicillin index %	19.93%	17.56%	18.61%	11.86%	11.40%

**Table 3:** The ten most consumed antibiotics for systemic use in Serbia, constituting more than 80% of total consumption from 2017 to 2021.

No.	2017 Antibiotic DID (%)	2018 Antibiotic DID (%)	2019 Antibiotic DID (%)	2020 Antibiotic DID (%)	2021 Antibiotic DID (%)
1	Amoxicillin 4.25 (20%)	Amoxicillin 4 (18%)	Amoxicillin 4.94 (19%)	Azithromycin 6.23 (21%)	Azithromycin 5.20 (15%)
2	Cephalexin 2.36 (11%)	Azithromycin 2.59 (11%)	Amoxicillin; clavulanic acid 3.66 (14%)	Amoxicillin 3.46 (12%)	Levofloxacin 4.49 (13%)
3	Amoxicillin; clavulanic acid 1.98 (9%)	Amoxicillin; clavulanic acid 2.56 (11%)	Azithromycin 2.84 (11%)	Amoxicillin; clavulanic acid 2.79 (10%)	Cefixime 4.18 (12%)
4	Ciprofloxacin 1.62 (8%)	Clarithromycin 1.69 (7%)	Cephalexin 2.29 (9%)	Levofloxacin 2.68 (9%)	Amoxicillin 3.93 (11%)
5	Doxycycline 1.36 (6%)	Ciprofloxacin 1.68 (7%)	Clarithromycin 1.65 (6%)	Cefixime 2.32 (8%)	Amoxicillin; clavulanic acid 3.46 (10%)
6	Azithromycin 1.36 (6%)	Doxycycline 1.64 (7%)	Ciprofloxacin 1.6 (6%)	Cephalexin 2.01 (7%)	Doxycycline 1.96 (6%)
7	Levofloxacin 1.25 (6%)	Levofloxacin 1.48 (7%)	Levofloxacin 1.5 (6%)	Doxycycline 1.98 (7%)	Cephalexin 1.85 (5%)
8	Clarithromycin 1.1 (5%)	Cefixime 1.30 (6%)	Doxycycline 1.47 (6%)	Ciprofloxacin 1.48 (5%)	Ciprofloxacin 1.75 (5%)
9	Cefixime 0.98 (5%)	Cephalexin 1.16 (5%)	Cefixime 1.4 (5%)	Clarithromycin 1.29 (4%)	Clarithromycin 1.46 (4%)
10	Sulfamethoxazole; trimethoprim 0.78 (4%)	Sulfamethoxazole; trimethoprim 0.71 (3%)	Sulfamethoxazole; trimethoprim 0.92 (3%)	Sulfamethoxazole; trimethoprim 0.82 (3%)	Ceftriaxone 1.13 (3%)

**Table 4:** MDR trend movements of selected bacteria in Serbia from 2017 to 2021.

%MDR Isolates of Total Number of Isolated Bacteria per Years						Linear Regression Analysis		
Bacteria	2017	2018	2019	2020	2021	Β	β	P
*K. pneumoniae*	64.3	58.6	65.1	69.2	71.7	2.540	0.799	0.107
*E. coli*	20.7	17.0	13.1	14.5	18.8	−0.630	−0.322	0.597
*P. aeruginosa*	51.2	56.0	56.4	61.4	53.3	0.960	0.395	0.510
*Acinetobacter* spp.	91.8	91.7	90.2	95.9	95.6	1.180	0.730	0.161

## Data Availability

Not applicable.
